# Effects of establishing a trauma center on the mortality rate among injured pediatric patients in Japan

**DOI:** 10.1371/journal.pone.0217140

**Published:** 2019-05-23

**Authors:** Takashi Muguruma, Chiaki Toida, Masayasu Gakumazawa, Naoki Yogo, Mafumi Shinohara, Ichiro Takeuchi

**Affiliations:** Department of Emergency Medicine, Yokohama City University Graduate School of Medicine, Yokohama, Japan; Oregon Health and Science University, UNITED STATES

## Abstract

**Introduction:**

It remains unclear whether trauma centers are effective for the treatment of injured pediatric patients. The aim of this study was to evaluate children’s mortality before and after the establishment a trauma center by using standard mortality ratios (SMR) and a modified observed-expected chart.

**Methods:**

This was a single center, retrospective chart review study that included injured pediatric patients (age <16 years) who were transported to our trauma center by the emergency medical services from 2012 to 2016 in Japan.

**Results:**

Our study included 143 subjects: 45 (31%) were preschoolers aged < 6 years, and 43 (30%) had an injury severity score (ISS) ≥ 16. After the trauma centers established, the number of patients increased (70% increase per month), as did the number of the patients with an ISS of 41–75. The percentage of indirect transportations was significantly higher in the trauma center than in the non-trauma center (49% vs. 28%; *p* < 0.05). The SMR was significantly lower in the trauma-center than in the non-trauma center (0.461 vs. 0.589; *p* < 0.05). The mean value of the modified observed-expected chart was significantly higher in the trauma-center than in the non-trauma center (4.6 vs. 2.3; *p* < 0.05). For the patients who were directly transferred to our center, the transfer distance was greater in the trauma-center than in the non-trauma center (6.8 vs. 6.2 km; *p* < 0.05). The time interval from hospital admission to initiation of computed tomography (15.5 vs. 33 minutes; *p* < 0.05) and to definitive care (44 vs. 64.5 minutes; *p* < 0.05) decreased in the after group compared to the non-trauma center.

**Conclusions:**

The results of our study revealed that the centralization of pediatric injured pediatric patients in trauma centers improved the mortality rate in this population in Japan.

## Introduction

Injuries are a leading cause of death and disability in Japanese youth aged 1 to 29 years of age [1–2]. It is essential that the transportation from the incident scene to the hospital by the emergency medical service (EMS), standardized in-hospital primary care, and definitive care are carried out quickly and appropriately to eliminate preventable deaths due to injuries [3]. Emergency medical centers play an important role in providing tertiary care for both the severely ill and injured patients.

However, previous studies have shown that there is a difference in in-hospital mortality of severely injured patients in each medical emergency center; designated trauma centers have superior survival rates for severely injured patient than the other undesignated centers [4–6]. Therefore, the Japanese Association for the Surgery of Trauma recommended the establishment of trauma centers in order to centralize severely injured patients and provide these patients with high-quality trauma care.

Owing to the relatively few trauma centers and the relatively low incidence of severely injured children in Japan, it remains unclear whether trauma centers are effective for the treatment of severely injured pediatric patients. In addition, when comparing the efficacy of medical care among institutions, several studies reported that two indicators, such as standard mortality ratio (SMR) and the modified observed-expected (O-E) chart, are useful [6–9]. The aim of this study was to evaluate children’s mortality before and after the establishment of one of the YCMTCs.by using SMR and a modified O-E chart.

## Materials and methods

### Study settings and patient population

This was a single center study, conducted retrospectively from January 1, 2012 to December 31, 2016 in Yokohama City University Medical Center (Yokohama, Japan). In 2016, Yokohama City had a population of 3.7 million, which included 466,984 children. Considering that one trauma center is established every 2 million people in the USA and UK [10–11], two trauma centers, named the Yokohama City Major Trauma Centers (YCMTCs), were first established by the local government in Yokohama City in 2014 [7]. Furthermore, a new “trauma bypass protocol” was introduced in the Yokohama City Fire Department, which allows the EMS to directly transfer severely injured patients to the trauma centers. The indication criteria of this new protocol was that the patient had an injury severity score (ISS) ≥16 or was suspected to have hemorrhagic shock. In order to initiate definitive care within 60 minutes of injury, the time interval between an emergency command notification and hospital arrival was planned to be within 45 minutes [12].

The Yokohama City University Medical Center is one of the two YCMTCs in the area. It provides trauma care to patients of all ages. This includes primary care in the emergency room and intensive care in the intensive care unit (ICU). There full-time staff comprises 26 people: 11 board-certified acute care physicians, 3 board-certified pediatricians, 5 board-certified intensive care physicians, 2 board-certified surgeons, 3 board-certified orthopedists, 1 board-certified plastic surgeon, and 1 board-certified neurosurgeon. Among these board-certified physicians, only 3 physicians had received training for the medical treatment of pediatric patients. We had 12 beds in the emergency ICU, 8 beds in the step-down unit, and 27 beds in the ward. The number of the patients transferred by the EMS to our center in 2015 was 1,667, of which 436 (26%) had been transferred due to severe trauma. Before transfer of a severely injured patient, an in-hospital trauma code was activated and preparations for emergency transfusions, emergency surgery, and interventional radiology (IVR) were initiated. After arrival at the hospital, the patient was immediately transferred to a primary care room where primary and secondary survey with computed tomography (CT) were performed. Whenever patients in unstable condition could not be transferred to the operating room, we performed emergency surgery, such as thoracotomy or laparotomy, in the primary care CT room.

**Fig 1:**
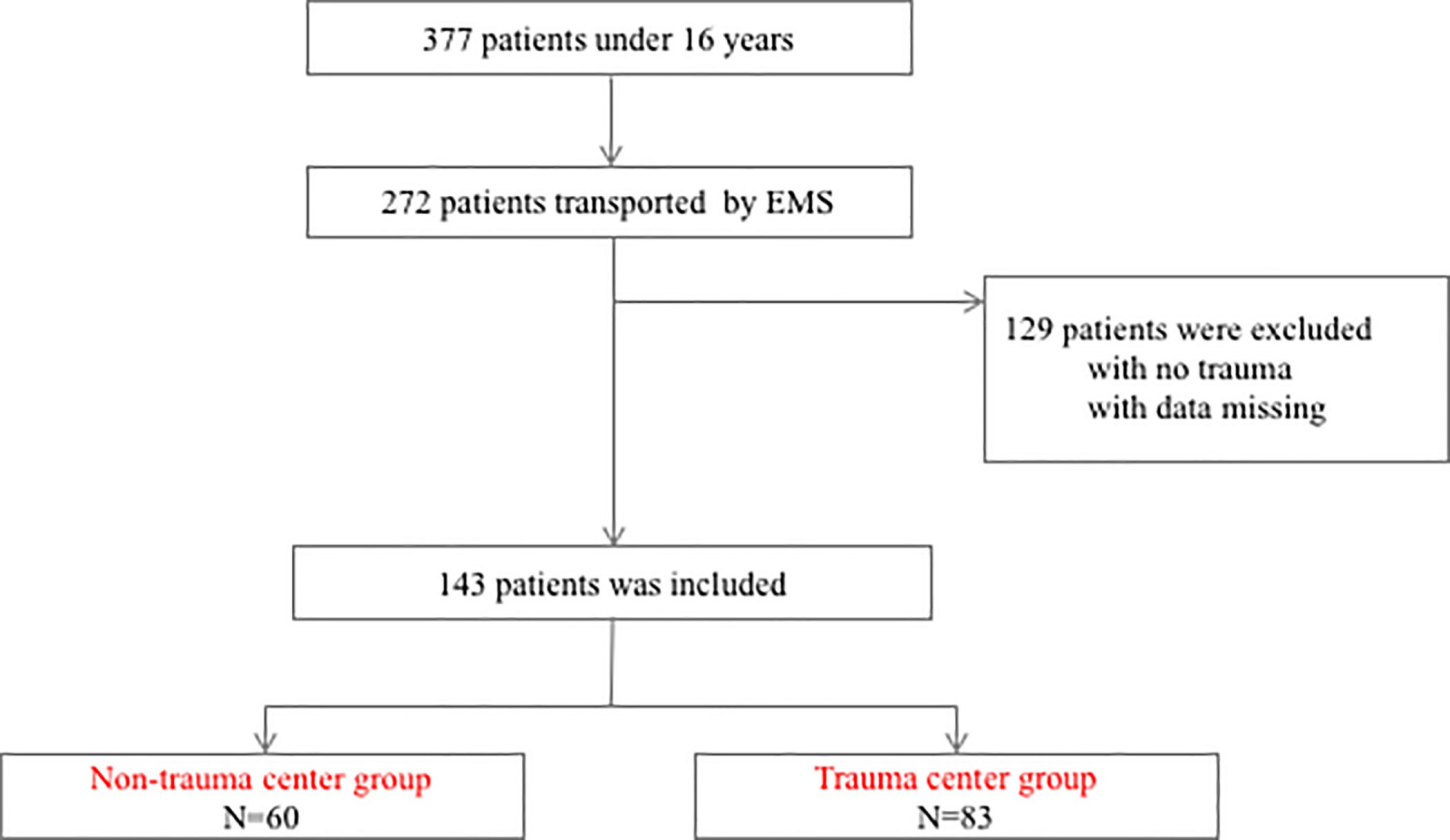
Flow diagram of the study population. One hundred and forty-three patients were enrolled and divided into either a non-trauma center (n = 60, admitted from January 2012 to September 2014) or a trauma-center (n = 83, admitted from October 2014 to December 2016).

The inclusion criteria for this study were as follows: (1) patients were aged under 16 years; (2) patients were transported by the EMS, including directly from the incident scene and from the other hospital; (3) patients suffered from the injury. Non-trauma patients and those for whom data was missing data were excluded from this study. Accordingly, 143 patients were enrolled and divided into a “non-trauma center group” (n = 60) that included patients admitted from January 1, 2012 to September 30, 2014, and an “trauma-center group” (n = 83) that included patients admitted from October 1, 2014 to December 31, 2016 (Fig 1). Although we modified our EMS transportation protocol in October 2014, we did not include a wash-out period because the new protocol was promptly enacted without a transition period.

### Data collection

The following data were collected for each patient: demographic variables [sex and age (years)], pre-hospital information [transfer by the EMS directly or indirectly from the incident scene, transfer distance (km)], clinical characteristics [mechanism of injury, injured organs (multiple system injury or single system injury with head injury), ISS, ISS category, and predicted survival rate (%)], in-hospital information [duration from admission to the initiation of CT or definitive care such as emergency operation, emergency IVR, and non-surgical treatment (minute)], and outcome information [predicted mortality rate upon admission to the ICU (%), duration of mechanical ventilation (days), ICU stay (days), hospital stay (days), in-hospital mortality rate (%), SMR, and excess survival]. Patients transported directly went straight from the incident site to our center with no intervening stops, whereas patients transported indirectly went from the incident site to a hospital and then to our center. Predicted survival rates were calculated by using the trauma and injury severity score (TRISS) method. Definitive care was defined as an invasive surgical intervention or if surgery was not required, critical care in the ICU. Invasive surgical interventions were performed after a primary or secondary survey in the emergency room, aiming to control of intracranial pressure, maintain of hemostasis, and repair of organs if necessary.

The primary endpoint was the mortality, such as standard mortality ratio and the modified observed-expected (O-E) chart, for all the injured pediatric patients who were transferred to our hospital by the emergency medical service (EMS). The secondary endpoint was the quality of the acute trauma care, especially from the hospital admission to the definitive care, for the injured pediatric patients who were transferred to our hospital directly from the injury site by EMS.

### Outcome measurements

As SMR and modified O-E chart were the primary outcome measures of this study, we compared these values before and after the establishment of our trauma center. As a secondary outcome measure, we examined patient characteristics. These included transfer time, the time interval between admission of a patient to the hospital and initiation of a CT scan, and whether interventional procedures were performed on children who were directly transferred to the trauma center.

The formula given below was used for calculating the SMR:

$$SMR=\frac{in-hospital\;mortality\;rate\;(\%)}{mean\;predicted\;mortality\;rate\;(\%)}$$

We used a modified O-E chart to calculate the number of excess survival cases, as shown in S1 Fig. Predicted survival rates (q) were calculated using the TRISS method and listed in a vertical bar chart in the decreasing order from left to right. A patient at position X with a predicted survival rate of qx was denoted as 1-qx if he/she survived or as–qx if he/she expired. The values per patient were added, and the cumulative values are shown in an O-E chart as a line graph prepared by linking neighboring values. A positive cumulative value indicated that more patients survived than expected, while a negative cumulative value indicated that more patients expired than expected.

Unlike the modified O-E chart, the original O-E chart did not include a vertical bar chart, and patients were arranged according to the date of hospital admission. By displaying the survival rates, the vertical bar chart allows the reviewer to easily discern the differences in outcomes in different conditions: favorable outcomes extend toward the top right, while unfavorable outcomes extend toward the bottom right. Hence, the modified O-E chart is more informative and reader-friendly than the original O-E chart.

### Statistical analysis

Continuous variables were expressed as medians with an interquartile range (25th–75th percentile); categorical variables were expressed as percentages. The Mann-Whitney U test and Fisher’s exact test were used for the analysis of continuous variables and categorical variables, respectively. Differences in means with 95% confidence intervals (CIs) were determined by using the independent t-test. In all statistical tests, a two-sided p value of less than 0.05 indicated statistical significance. Analyses were performed using STATA SE software, version 12.1 (College Station, TX, USA).

### Ethics statement

This retrospective chart review was approved by the Independent Ethics Committee of Yokohama City University School of Medicine to assure that patient confidentiality was maintained. Requirement of informed consent from the patients was waived due to the observational nature of the study design.

## Results

A total of 377 children were transferred to our center. Of the 272 patients transferred by the EMS, 143 met the inclusion criteria. The study population included 107 (75%) males and 45 (31%) preschoolers aged less than 6 years. The median age of the study subjects was 8 years (interquartile range: 4–11 years old). Eighty-five (59%) patients transferred by the EMS directly from the injury site. Forty-three (30%) patients had an ISS ≥16 and 86 (60%) required emergency surgery or IVR within 24 hours of arrival to the hospital. The emergency surgeries performed included craniotomy or trepanation (16 patients, 11%), thoracotomy or laparotomy (11 patients, 8%), open reduction and internal fixation (26 patients, 18%), and surgeries classed as others (30 patients, 21%). Some patients underwent more than one type of surgery and these patients were represented across different groups based on the type of surgery. Ninety (63%) patients were admitted to the ICU. The trauma-center than in the non-trauma center.

### SMR and modified O-E chart of all patients between the non-trauma center and trauma-center

A comparison of the two groups is shown in Table 1. The mean number of patients transferred to our center increased by 70% following the establishment of the YCMTCs; 3.1 and 1.8 patients were admitted per month for after and before the establishment of the YCMTCs, respectively. The percentage of indirect transportations was significantly higher in the trauma center than in the non-trauma center (49% vs. 28%; *p* < 0.05). Moreover, the percentage of the patients with ISS ≥16 who were transferred from the other hospital was significantly higher in the trauma center than in the non-trauma center (17% vs. 3%; *p* < 0.05).

**Table 1:** Patient characteristics and outcomes between the non-trauma center and trauma-center.

Variable	Non-trauma center group (n = 60)	Trauma center group (n = 83)	P value	Difference in mean value / ratio	95% CI
Male, n (%)	50 (83)	57 (69)	<0.05	14.6%	(12.9−16.2)
Age in year, (median, IQR)	8 (5.5–11.5)	7 (4–11)	0.21	11.7	(9.6−13.8)
Transportation from the injury site, n (%)	43 (72)	42 (51)	<0.05	21%	(19.1−22.9)
Transportation from another hospital, n (%)	17 (28)	41 (49)	<0.05	-21%	(-19.1−-22.9)
patients with Injury Severity Score ≧ 16	2 (3)	14 (17)	<0.05	-13.5%	(61.7−98.4)
Mechanism of injury					
Penetrating injury, n (%)	2 (3)	3 (4)	0.93	-0.3%	(-1.0−4.1)
Blunt injury, n (%)	58 (97)	80 (96)	0.93	0.3%	(-0.4−1.0)
traffic accident, n (%)	30 (50)	33 (40)	0.22	10.3%	(8.3−12.2)
fall, n (%)	15 (25)	13 (16)	1.17	9.4%	(7.8−10.9)
Turnover, n (%)	7 (12)	13 (16)	0.50	-4%	(-5.3−-2.7)
Injury Severity Score, (median, IQR)	9 (4–16)	9 (4–17)	0.79	-1.6	(-2.3−-1.1)
Injury Severity Score category					
16–24, n (%)	7 (12)	10 (12)	1.00	-0.4%	(-1.7−0.9)
25–40, n (%)	8 (13)	10 (12)	0.81	1.3%	(0−2.6)
41–75, n (%)	1 (2)	7 (8)	<0.05	-6.8%	(-7.6−-6)
Predicted survival rate, %, (median, IQR)	99.4 (98.5–99.6)	99.4 (98.1–99.7)	0.43	4.7%	(3.8−5.6)
Tertiary Care					
emergency operation, n (%)	26 (43)	47 (57)	0.12	-13.3%	(-15.2−-11.4)
craniotomy or trepanation, n (%)	4 (7)	12 (14)	0.18	-7.8%	(-8.9−-6.6)
thoracotomy or laparotomy, n (%)	4 (7)	7 (8)	0.76	-1.0%	(-1.9−-1.0)
open reduction and internal fixation, n (%)	10 (17)	16 (19)	0.83	-2.6%	(-4.1−-1.1)
others, n (%)	8 (13)	22 (27)	0.06	-13.2%	(-14.7−-11.7)
emergency Interventional Radiology, n (%)	5 (8)	8 (10)	0.79	-1.3%	(-2.4−-0.2)
Duration of mechanical ventilation, days, (median, IQR)	0 (0–2)	1 (0–3)	<0.05	-1.3	(-1.5−-1.1)
Duration of ICU stay, days, (median, IQR)	3 (2–4)	2 (2–6)	0.80	-3.7	(-4.3−-3.2)
Duration of hospital stay, days, (median, IQR)	7 (4–17)	8 (4–19)	0.62	-4.5	(-5.3−3.7)
Standard mortality ratio	0.589	0.461			
In-hospital actual mortality, n (%)	2(3)	4 (5)	0.662	-1.5%	(-2.3−-0.7)

**Fig 2:**
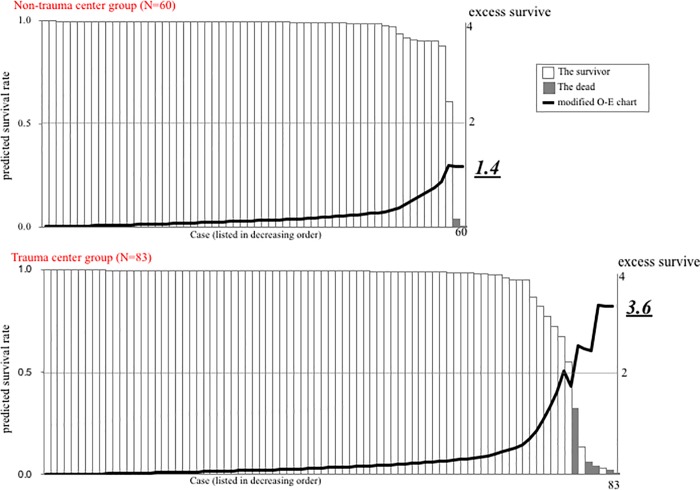
The modified observed-expected (O-E) chart between the non-trauma center and trauma-center. Excess survival was calculated using the modified O-E chart. The number of patients who exceeded survival was 1.4 per 60 transfers in the non-trauma center and 3.6 per 83 transfers in the trauma center.

There was no difference in median ISS between the non-trauma center and trauma-center. However, the number of patients with an ISS of 41–75 was significantly higher in the trauma-center than in the non-trauma center (8% vs. 2%; *p* < 0.05). The SMR was significantly lower in the trauma vs. non-trauma center (0.461 vs. 0.589; *p* < 0.05). The mean value of modified O-E chart was significantly higher in the trauma center than in the non-trauma center (4.6 vs. 2.3; *p* < 0.05). Two patients in the non-trauma center had a predicted survival rate <50%, both of whom died (Fig 2). Seven patients in the trauma center had a predicted survival rate of <50%, of which 2 survived. Survival rates for the 2 survivors in the trauma center were 13.4% and 3.1%. The Pediatric Cerebral Performance Category for one of the patients was 2. None of the patients with a predicted survival rate >50% died in either group. Excess survival was calculated using the modified O-E chart. The number of patients who exceeded their predicted survival was 1.4 per 60 transfers in the non-trauma center and 3.6 per 83 transfers in the trauma center (Fig 2). For every 100 patients transferred to our center during the survey period, 2.3 and 4.4 patients exceeded their predicted survival in the non-trauma center and trauma center, respectively.

### Characteristics of patients directly transferred to our center

The characteristics of patients directly transferred to our center are shown in Table 2. For patients with an ISS ≥16, the transfer distance was significantly greater in the after than in the non-trauma center [6.8 (5.8–12.4) km vs. 6.2 (3.3–8.3) km; *p* < 0.05, intercept: 0.6 km, 95% CI: –1.3–-0.4]. The time interval from hospital admission to the initiation of CT scanning was significantly lower in the after than in the non-trauma center [15.5 (11–19) minutes vs. 32 (24–38) minutes; *p* < 0.05, intercept: 12.7 minutes, 95% CI: 9.5–15.9]. The time interval from hospital admission to the initiation of definitive care was also significantly lower in the trauma center than in the non-trauma center [44 (36–54) minutes vs. 64.5 (55–81) minutes; *p* < 0.05, intercept: 23.1 minutes, 95% CI: 15.3–30.8]. Definitive care was initiated within 60 minutes of hospital admission for all patients in the trauma center.

**Table 2:** The results of the outcomes measures for the patients who were directly transferred from the injury site.

Variable	Non-trauma center group (n = 43)	Trauma center group (n = 42)	P value	Difference in mean value / ratio	95% CI
Injury Severity Score, (median, IQR)	9 (4–17)	5 (1–17)	0.37	-0.15	-1.0 to 0.7
Injury Severity Score category					
16–24, n (%)	6 (14)	4 (10)	0.74	4.4%	(2.2 to 6.5)
25–40, n (%)	7 (16)	4 (10)	0.52	6.7%	(4.5 to 8.9)
41–75, n (%)	1 (2)	5 (12)	0.11	-9.6%	(-11.2 to -8.0)
Predicted survival rate, %, (median, IQR)	99.3 (97.2–99.6)	99.4 (95.9–99.7)	0.47	4.8%	(3.2 to 6.3)
The transportation distance (km)					
All patient	6.3 (3.3–8.3)	6.4 (5.6–10.9)	0.20	-0.1	(-1.7 to -1.1)
patients with Injury Severity Score ≧ 16	6.2 (3.3–8.3)	6.8 (5.8–12.4)	<0.05	-0.6	(-1.3 to -0.4)
Time interval from admission to the beginning of CT, minute, (median, IQR)					
All patient	33 (26–838)	18 (13–27)	<0.05	13.3	(12.0 to 14.8)
patients with Injury Severity Score ≧ 16	33 (24–38)	15.5 (11–19)	<0.05	12.7	(9.5 to 15.9)
Time interval from admission to the beginning of tertiary care, minute, (median, IQR)					
All patient	67.5 (56–82.5)	46 (39–54)	<0.05	25.7	(23.1 to 28.3)
patients with Injury Severity Score ≧ 16	64.5 (55–81)	44 (36–54)	<0.05	23.1	(15.3 to 30.8)
In-hospital actual mortality, n (%)	2 (5)	2 (5)	1.00	-0.1	(-1.5 to 1.3)

### Discussion

In this study, we evaluated an effect of the establishment of a trauma center on in-hospital mortality among injured pediatric patients. Our study suggests that trauma centers can provide the definitive care for severely injured patients quickly and effectively as patients are centralized. Our findings are in line with those of previous studies, in which there is a relationship between better outcomes and the centralization of severely injured patients [13–14]. Moreover, this study showed the very interesting result that the number of indirect transfers increased to a greater extent than that of direct transfers because we introduced the only prehospital protocol, which was mainly used in the prehospital phase by the EMS staff.

It is difficult to explain why only shortening of the trauma care in the acute care phase improves the outcome of the injured pediatric patients. We suspect that the following are the reasons to improve the trauma care for injured pediatric patients: (1) newly developed prehospital protocols contributed to the centralization of the injured patients; (2) improvement in the treatment environment, such as the establishment of primary care CT rooms and in-hospital trauma codes, contributed to provide quick and effective trauma care; and (3) acquirement of experience of trauma care for pediatric patients contributed to the skill of the trauma team. As several practical approaches, shortening of the trauma care in the acute care phase reduces the mortality of the injured pediatric patients in the trauma center. The time interval between the occurrence of an injury and the start of definitive care is a key determinant of the outcome; severely injured patients requiring urgent surgical intervention or IVR should be treated within 1 hour of injury [14–15]. However, the relationship between timely trauma care and a better outcome is not simple. For example, in previous studies, rapid delivery of medical care at trauma centers improved outcomes in severely injured children only when the quality of trauma care was high [16–17]. In the present study, we only examined the time interval from hospital admission to the beginning of CT and definitive care in order to evaluate the quality of trauma care in the acute care phase. It is really necessary to examine the quality of medical care in several phases from the prehospital care phase to recovery care phase in order to evaluate the quality of the trauma care per facilities. As a next step, it is necessary to investigate all kinds of medical care offered to the pediatric patients from the time of an accident to discharge from the hospital.

We considered the reason why this study has shown the following: (1) in terms of direct transfer by the EMS, the distance between the pick-up location and our trauma center differed significantly but not substantially (only 0.6 km) for before and after the establishment of the YCMTCs and (2) the number of indirect transfers increased to a greater extent. First, according to the Fire and Disaster Management Agency in Japan, children with non-severe injuries account for 35% of patients transported by the EMS, whereas children with severe injuries account for only 0.2% [18]. As the incidence of severe injuries in children is low, the number of children with severely injuries who were transferred directly from the incident scene by the EMS was presumably low. It is thought that the prehospital protocol error might have occurred by the EMS staff because the distance between the pick-up location and our trauma center differed significantly, but not substantially (by only 0.6 km) before and after the establishment of the YCMTCs. In this study, however, we cannot evaluate the validity of the newly developed prehospital protocol. Further examination of this will be necessary in the future. Furthermore, we should pay greater attention to the result that the number of indirect transfers increased to a greater extent. This result showed that the establishment of the inter-hospital transportation system from the primary hospital to the trauma center should be essential to improve outcomes, especially in severely injured pediatric patients. This indicates that a high number of severely injured children were transferred from remote locations, in which case more attention was paid to improve their outcomes. When severely injured children are transported from a remote location, using helicopter emergency medical services or a doctor’s car might be beneficial because it would reduce the time from providing definitive care and advanced interventions such as airway management in the prehospital setting [19]. Furthermore, previous studies have identified the following key factors for improving outcomes in cases involving multiple hospitals: (1) supervision of inter-hospital transportation by teams of specialists while stabilizing the patients at the primary hospital [20] and (2) incorporating input from specialists in hospitals with ICUs regarding appropriate interventions and transportation [21]. In the future, to improve the quality of medical care for severely injured, indirectly transferred patients, teams of specialists should be dispatched from the trauma center to the scene of the accident or to the primary hospital for early-stage interventions.

We note some study limitations. First, it was a retrospective analysis from a single institution. So, the number of cases for this study was small and there was a difference in the severity of cases between two groups. Second, outcomes for patients with severe injuries cannot be compared between adults and children. Originally, there was no difference in medical care procedures between severely injured children and adults [22]. Although children differ both anatomically and physiologically from adults, the introduction of child-specific treatments tools will further improve the outcomes of severely injured patients regardless of age [22]. Furthermore, there was a difference in the number of medical personnel that specialized in pediatric critical care between the two study groups. The two physicians with pediatric critical care experience began working at our center several months prior to the establishment of our institution as a trauma center; this may have biased the health outcomes of pediatric patients. Additionally, because the quality of every trauma care including surgery and intensive care was not able to evaluated in this study (e.g., proficiency degree of operator and medical stuffs who treated the injured pediatric patients), unfortunately, uncertain who to make of the ultimate conclusion of decrease time to scanning as well as lower mortality given the increase in the most severely injured children is not significant. Therefore, in the future, we intend to conduct an additional and detailed survey of all emergency centers, including two trauma centers in our region, to verify the ultimate effects of trauma center establishment on the mortality rate in pediatric trauma patients.

## Conclusion

Our study suggests that the centralization of pediatric trauma patients to trauma centers improved the morality of injured pediatric patients. In the future, we can reduce mortality rates of severely injured pediatric patients in a whole region by centralizing patients from a larger area in the designed trauma center.

## Supporting information

S1 Fig A modified O-E chart to calculate the number of excess survival cases. Predicted survival rates (q) were calculated using the trauma injury and injury severity score method and listed in a vertical bar chart in the decreasing order from left to right (S1A Fig). A patient at position X with a predicted survival rate of qx was denoted as 1-qx if he/she survived or as–qx if he/she expired. The values of each patient were added and the cumulative values are shown in an O-E chart as a line graph prepared by linking neighboring values (S1B Fig). A positive cumulative value indicated that more patients survived than expected, while a negative cumulative value indicated that more patients expired than expected.
(TIFF)

S1 Dataset The Minimal data set supporting information file. (PDF)
