# Supplementary figures and images for: Effects of establishing a trauma center on the mortality rate among injured pediatric patients in Japan
Source: PLoS One. 2019 May 23;14(5):e0217140. doi: 10.1371/journal.pone.0217140 (PMC6532880; doi:10.1371/journal.pone.0217140)

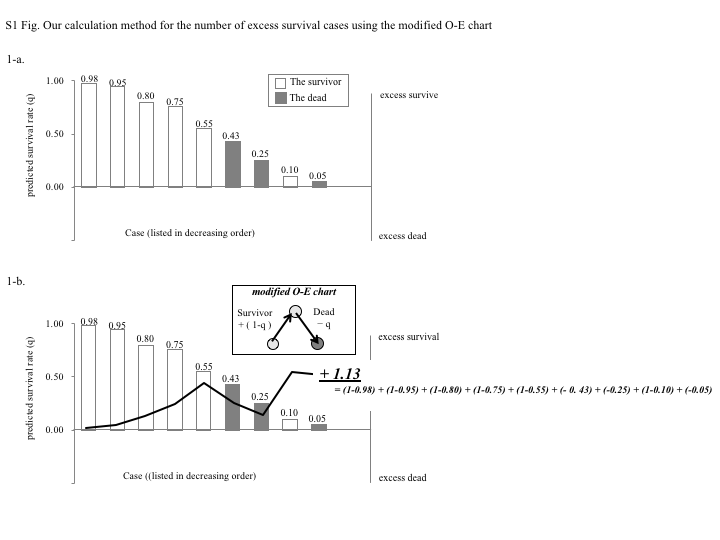

Supplement: S1 Fig — Predicted survival rates (q) were calculated using the trauma injury and injury severity score method and listed in a vertical bar chart in the decreasing order from left to right (S1A Fig). A patient at position X with a predicted survival rate of qx was denoted as 1-qx if he/she survived or as–qx if he/she expired. The values of each patient were added and the cumulative values are shown in an O-E chart as a line graph prepared by linking neighboring values (S1B Fig). A positive cumulative value indicated that more patients survived than expected, while a negative cumulative value indicated that more patients expired than expected. (TIFF) [file pone.0217140.s001.tiff]
