# Supplementary material for: Effects of establishing a trauma center on the mortality rate among injured pediatric patients in Japan
Source: PLoS One. 2019 May 23;14(5):e0217140. doi: 10.1371/journal.pone.0217140 (PMC6532880; doi:10.1371/journal.pone.0217140)
Supplement: S1 Dataset — (PDF) [file pone.0217140.s002.pdf]

Trauma center group	male	Duration of ICU stay	Duration of hospital stay	Injury severity score	Predictive survaival rate	death	Blunt injury	Penetrating injury	emnergency operation	emnergency interventional radiology	Transportation from the injury site
0	1	0	0	57	0.001	1	1	0	1	0	1
0	1	17	58	26	0.976	0	1	0	1	1	0
0	1	3	15	9	0.989	0	1	0	1	1	0
0	1	3	8	17	0.938	0	1	0	0	0	1
0	0	2	2	16	0.990	0	1	0	0	0	1
0	0	3	7	16	0.990	0	1	0	1	1	1
0	1	0	0	34	0.036	1	1	0	0	0	1
0	1	3	6	26	0.901	0	1	0	0	0	1
0	1	4	4	21	0.984	0	1	0	1	0	1
0	1	3	8	17	0.989	0	1	0	0	0	0
0	0	11	24	33	0.915	0	1	0	1	0	1
0	1	6	23	26	0.901	0	1	0	1	0	1
0	1	13	36	26	0.901	0	1	0	1	0	1
0	1	5	15	17	0.989	0	1	0	1	0	1
0	1	9	30	29	0.608	0	1	0	0	0	1
0	1	10	22	26	0.877	0	1	0	1	0	1
0	1	2	7	19	0.987	0	1	0	0	0	1
0	0	2	2	5	0.992	0	1	0	0	0	0
0	0	0	1	1	0.996	0	1	0	0	0	1
0	1	0	0	1	0.998	0	1	0	0	0	1
0	1	3	4	9	0.993	0	1	0	1	0	1
0	1	0	2	4	0.997	0	1	0	1	0	0
0	1	2	5	1	0.997	0	1	0	0	0	0
0	0	1	2	10	0.994	0	1	0	0	0	0
0	1	3	6	5	0.996	0	1	0	1	0	1
0	1	2	3	1	0.996	0	1	0	0	0	1
0	1	0	0	1	0.998	0	0	1	0	0	1
0	1	2	3	4	0.908	0	1	0	0	0	1
0	1	0	6	2	0.997	0	1	0	1	0	1

0	1	3	17	11	0.985	0	1	0	0	0	1
0	1	0	2	5	0.995	0	1	0	0	0	1
0	1	2	2	5	0.996	0	1	0	0	0	1
0	1	0	3	10	0.992	0	1	0	0	0	1
0	0	4	4	9	0.994	0	1	0	0	0	1
0	1	2	2	5	0.994	0	1	0	0	0	1
0	1	1	1	4	0.996	0	1	0	0	0	0
0	1	3	12	4	0.995	0	1	0	0	0	0
0	1	0	2	1	0.994	0	1	0	0	0	1
0	1	4	9	9	0.994	0	1	0	1	1	0
0	1	2	3	10	0.984	0	1	0	0	0	1
0	0	0	2	10	0.994	0	1	0	1	0	0
0	1	1	4	9	0.994	0	1	0	0	0	0
0	0	2	7	9	0.994	0	1	0	0	0	0
0	1	2	4	5	0.996	0	1	0	1	0	1
0	1	0	4	1	0.996	0	1	0	0	0	1
0	1	2	5	12	0.984	0	1	0	1	0	1
0	1	2	2	9	0.993	0	1	0	0	0	1
0	1	3	9	9	0.994	0	1	0	0	0	0
0	0	2	21	1	0.996	0	1	0	1	0	0
0	1	3	8	11	0.991	0	1	0	0	0	0
0	1	0	2	4	0.997	0	1	0	0	0	1
0	1	2	25	10	0.994	0	1	0	1	0	1
0	1	2	23	10	0.994	0	1	0	1	0	1
0	1	0	3	9	0.994	0	1	0	0	0	1
0	1	0	2	6	0.995	0	1	0	0	0	1
0	1	4	21	5	0.997	0	1	0	1	0	1
0	1	3	11	10	0.994	0	1	0	1	1	0
0	1	0	2	10	0.994	0	1	0	1	0	1
0	1	0	15	4	0.996	0	1	0	1	0	1
0	1	2	13	4	0.972	0	0	1	1	0	1
1	1	70	109	59	0.003	0	1	0	1	1	0
1	1	2	2	32	0.042	1	1	0	1	0	0
1	1	2	25	9	0.994	0	1	0	1	0	0
1	1	0	0	36	0.031	0	1	0	1	0	1
1	1	1	1	41	0.020	1	1	0	1	0	1
1	1	12	16	25	0.973	0	1	0	1	0	0
1	0	0	4	17	0.986	0	1	0	0	0	1
1	1	0	24	17	0.998	0	1	0	0	0	0
1	1	2	8	16	0.987	0	1	0	0	0	0

1	1	7	7	16	0.821	0	1	0	0	0	0
1	1	3	17	16	0.987	0	1	0	1	0	0
1	0	13	31	21	0.959	0	1	0	1	0	1
1	0	8	8	50	0.061	1	1	0	1	0	0
1	0	6	6	33	0.948	0	1	0	0	0	0
1	1	23	23	17	0.134	0	1	0	0	0	1
1	0	8	19	43	0.325	1	1	0	1	1	1
1	1	3	5	26	0.976	0	1	0	1	0	0
1	0	3	5	17	0.989	0	1	0	0	0	0
1	0	50	89	26	0.771	0	1	0	1	0	1
1	0	3	8	26	0.951	0	1	0	0	0	1
1	1	2	4	17	0.989	0	1	0	0	0	1
1	0	4	19	27	0.949	0	1	0	1	1	0
1	1	2	9	16	0.990	0	1	0	1	0	0
1	1	27	73	50	0.550	0	1	0	1	1	1
1	1	6	85	30	0.866	0	1	0	1	0	1
1	1	2	23	25	0.978	0	1	0	1	0	0
1	1	24	59	41	0.721	0	1	0	1	1	1
1	1	15	64	41	0.672	0	1	0	1	1	1
1	0	0	6	5	0.996	0	1	0	1	0	1
1	1	2	3	13	0.992	0	1	0	0	0	0
1	1	2	2	2	0.996	0	1	0	1	0	0
1	0	0	8	1	0.996	0	1	0	1	0	1
1	1	0	4	1	0.998	0	1	0	1	0	0
1	1	0	0	0	0.997	0	1	0	0	0	1
1	0	0	0	0	0.997	0	1	0	0	0	1
1	1	0	9	4	0.997	0	1	0	1	0	1
1	1	0	2	1	0.997	0	1	0	1	0	1
1	0	1	2	1	0.997	0	1	0	0	0	1
1	1	2	2	5	0.989	0	1	0	0	0	1
1	1	1	8	1	0.997	0	0	1	1	0	1
1	0	0	0	1	0.997	0	0	1	0	0	0
1	1	2	5	9	0.994	0	1	0	0	0	0
1	1	6	9	9	0.993	0	1	0	0	1	0
1	1	0	0	9	0.997	0	1	0	0	0	0
1	1	0	0	1	0.996	0	0	1	0	0	1
1	1	0	0	1	0.997	0	1	0	1	0	1
1	0	0	2	5	0.989	0	1	0	1	0	1
1	1	2	6	1	0.996	0	1	0	1	0	0
1	1	2	7	14	0.991	0	1	0	1	0	1

1	1	1	2	5	0.996	0	1	0	0	0	1
1	1	6	7	4	0.990	0	1	0	0	0	1
1	0	0	0	1	0.998	0	1	0	0	0	1
1	1	0	6	10	0.992	0	1	0	1	0	1
1	1	0	2	8	0.995	0	1	0	0	0	0
1	0	0	3	5	0.996	0	1	0	1	0	0
1	1	0	5	4	0.998	0	1	0	1	0	0
1	0	0	2	4	0.997	0	1	0	0	0	0
1	1	6	18	10	0.983	0	1	0	1	0	1
1	0	0	3	9	0.994	0	1	0	0	0	0
1	0	0	2	9	0.994	0	1	0	0	0	0
1	0	0	4	4	0.998	0	1	0	1	0	0
1	1	0	2	4	0.997	0	1	0	0	0	0
1	1	0	0	4	0.996	0	1	0	0	0	0
1	0	2	11	14	0.991	0	1	0	1	0	0
1	1	2	6	11	0.991	0	1	0	1	0	0
1	1	0	26	5	0.995	0	1	0	1	0	1
1	1	2	2	5	0.994	0	1	0	1	0	1
1	1	0	0	4	0.996	0	1	0	0	0	0
1	1	2	6	4	0.996	0	1	0	0	0	0
1	1	0	0	2	0.998	0	1	0	1	0	0
1	1	0	5	10	0.994	0	1	0	1	0	1
1	0	4	6	9	0.994	0	1	0	0	1	0
1	1	0	0	1	0.997	0	1	0	1	0	1
1	1	2	9	10	0.994	0	1	0	0	0	0
1	1	2	3	6	0.997	0	1	0	1	0	1
1	0	2	4	2	0.996	0	1	0	0	0	1
1	1	0	2	1	0.996	0	1	0	0	0	1
1	0	0	0	1	0.998	0	1	0	0	0	1
1	1	0	3	5	0.996	0	1	0	1	0	1
1	1	0	0	1	0.998	0	1	0	1	0	1
1	0	1	9	14	0.981	0	1	0	1	0	1
1	1	1	2	9	0.994	0	1	0	0	0	0
1	1	0	6	1	0.998	0	1	0	1	0	0
